# Geochemical balance of the mineral fraction and statistical analyzes of the Ebolowa municipal lake’s sediments (central Africa): Implication for early diagenesis process

**DOI:** 10.1016/j.heliyon.2023.e17617

**Published:** 2023-06-24

**Authors:** Akono Daniel Florent, Bokanda Ekoko Eric, Bisse Salomon Bertrant, Belinga Belinga Cedric, Menanga Tokouet Renaud, Tonye Marie-Diane, Nzesseu Nandjou Valentino, Biani Nya Estelle Diane, Kouakam Cedric, Ngono Anaba Leon Vital, Mouhamed Amin Nsangou, Ekomane Emile

**Affiliations:** a Department of Earth Sciences, University of Yaounde I, PO Box 812, Yaounde, Cameroon; b Department of Geology, University of Buea, PO Box 63, Buea, Cameroon; c School of Geology and Mining Engineering, PO Box 115, Meiganga, Cameroon

**Keywords:** Geochemical balance, Early-diagenesis, Lake, Ebolowa

## Abstract

The Ebolowa Municipal Lake (EML) (South Cameroon) in order to identify the early diagenesis processes taking place in the lake and the factors influencing them. To this end, 21 samples were collected. In situ, hydrogen potential, redox potential, conductivity, dissolved oxygen content, and turbidity were measured. In the laboratory, the samples were subjected to mineralogical analysis by X-ray diffraction, geochemical analysis by X-ray fluorescence and ICP-MS, and statistical analysis. The coefficient of variation (Qi) was calculated from the geochemical data. In the water column, OD > 2 mg/L, pH > 7 and Eh < 0 mV. In sediments: pH < 7, Eh values are lower. The contents of 2.08 ≤ TOC ≤ 12.65%. The mineralogical procession consists of quartz, kaolinite, gibbsite, goethite, and siderite. The latter is only present in the EML. The sediments are dominated by SiO_2_ (60.44–89.47%), Al_2_O_3_ (6.55–18.17%), and Fe_2_O_3_ (1.15–6.21%). The Qi values range from 0.73 to 2.31. The Mn/Fe ratio values are below 0.40. Qi > 1 for Al, Fe, Mn, Mg, K, Na, P, Ni, Co, Zn, Pb, Cd, Cu, Ba, and V, and Qi < 1 for Si; Qi = 1 for Ca. The hierarchical cluster analysis shows two groups: the first one includes the samples from the central and western parts, while the second one includes those from the eastern and southern parts of the lake. The water column is subject to oxic conditions, while the sediments are anoxic. The rapid consumption of oxygen is due to organic mineralization, which is the main diagenesis observed in the lake. This phenomenon is more accentuated in the western part of the lake.

## 1 Introduction

Lakes are extremely diverse bodies of water. They are home to significant biodiversity and are at the heart of essential stakes for mankind like water source, fishing, and tourism. They occupy nearly 1.8% of the world's surface and represent about 0.19% of the volume of water in the hydrosphere [1]. Lakes are high-resolution archives of local and regional change, allowing paleoenvironmental and soil-climate reconstructions to be made [2]. The composition of lake environments depends mainly on exogenous factors such as the nature of the bedrock, climate, and catchment characteristics, but also on autochthonous factors. Indeed, lakes are very dynamic systems in which a number of processes take place [3]. These processes, known as early diagenesis, include sorption phenomena (e.g.: precipitation, absorption, and dissolution, etc.) and the mineralization of organic matter [4]. They take place along the water column up to the first meter of the bottom sediments, thus modifying the composition of the particles that are deposited. Early diagenesis can indeed cause the release of certain elements associated with the solid phase of the sediments or the precipitation and subsequent deposition of ions present in the water column. This can increase or decrease the concentration of various elements, including those that are dangerous for the environment such as trace metals (cadmium, lead, copper, zinc, etc.), thus contributing to the pollution of the water column, the acceleration of the eutrophication phenomenon, or the formation of authigenic minerals [[5], [6], [7], [8], [9]]. For this reason, the understanding of diagenetic phenomena in aquatic environments has been the subject of several studies [[10], [11], [12], [13]]. These works have shown that early diagenesis is influenced by the variation of redox conditions, which has a great influence on the physicochemical and biological parameters of the environment. Several studies [10,12] have shown that dissolved oxygen content, redox potential, hydrogen potential, and iron and manganese contents are good markers of redox and paleo-redox conditions. These studies are most often based on the analysis of the water column, the organic and inorganic fractions of the sediment, and the biological fraction of the environment [[14], [15], [16], [17]]. According to the work of [18], rivers are flowing environments, while lakes are stagnant environments. This difference in property makes lakes favorable environments for early diagenesis. In sub-Saharan Africa, some lakes are fed by rivers and are the subject of several studies. However, these studies are mainly concerned with palaeoenvironmental reconstructions, provenance, paleoweathering conditions, and t pollution level. Few studies have focused on early diagenesis process and its influence on sediments composition [[19], [20], [21], [22]]. The Ebolowa Municipal Lake (EML) is fed by two rivers. This makes it a favorable environment for processes associated with early diagenesis. Also, the work of [21,22] showed that there is a difference in the geochemical composition of the sediments of the EML compared to those of its tributaries, although they come from the same sources and have undergone the same type of alteration. Similarly, the lake has been undergoing accelerated eutrophication for several years, especially in its Western part. The present work aims to carry out a geochemical assessment of the EML, in order to (i) determine the redox conditions prevailing in the water column and bottom sediments; (ii) identify the diagenesis processes that take place in this lake; and (iii) quantify the influence of these processes on the composition of the EML sediments.

## 2 Study area

**Fig. 1:**
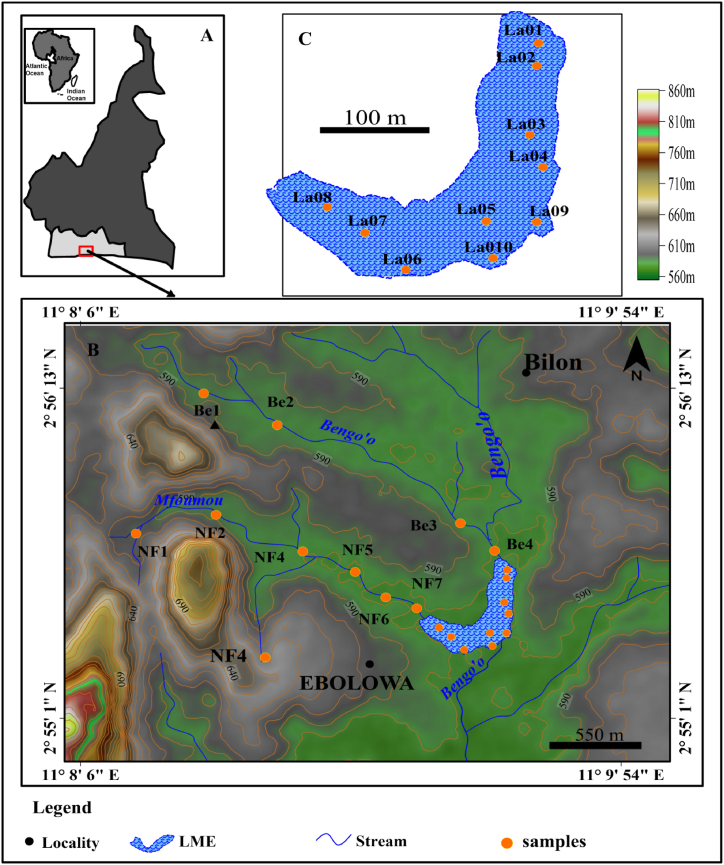
Location map of the study area (a) map of Cameroon; (b) global map of the study area; (c) map of the EML.

The municipal lake of Ebolowa is located in the regional capital of South Cameroon. It is localized between 2°52′ and 2°58′ north latitude and 11°06′ and 11°12′ east longitude in southern Cameroon. The climate of the area is equatorial Guinean. This is characterized by the alternation of four seasons, including two dry seasons and two rainy seasons. The average annual temperatures and rainfall are, respectively, 24.8 °C and 1673 mm. Ferralitic soils and 93 hydromorphic soils coexist in the region. The vegetation is a dense, degraded forest and shrubs in urban areas. The study area belongs to the Ntem complex [23]. It is composed lithologically of the Charnockite suite and gneisses [24,25]. The Municipal Lake of Ebolowa was created in 1962 as a recreational area in the country. It has a perimeter of 2.13 km and a surface area of 13.25 ha [26]. The Bengo'o and Mfoumou streams supply it with water. Before spilling into the lake, the Bengo'o Stream flows for roughly 10 km. It runs through the Mikongo, Bilon, Angalé, and Ekombité districts' marshy areas. The Mfoumou Stream originates in the New-Bell neighborhood. It flows for roughly 5 km through the districts of New-Bell I, New-Bell II, and New-Bell III, the Oyenga Market, Ekombité, and the central market before flowing into the lake (Fig. 1a–c).

## 3 Methodology

### 3.1 Field work

Twenty-one superficial sediment samples (less than 10 cm thick) were collected from the lake and its two tributaries (Fig. 1). This was made possible by using an Eckman bucket for the lake and a plastic trowel for the tributaries. For each sampling point, the physicochemical parameters of the water were measured using a HANNA H192982/20 multiparameter device. These were: conductivity (Cs), salinity (Sal), dissolved oxygen content (DO), temperature (T), hydrogen potential (pH), turbidity (Turb), and redox potential (Eh). For sediments, pH and Eh were measured according to the method described by Ref. [22]. The collected samples were packed in plastic bags with a supernatant of water from each sampling point.

### 3.2 Laboratory analysis

In the laboratory, the samples were described according to three criteria: color, texture, and detrital content. The color of the different samples was determined by comparison with the Munsell soil color chart. The water content was measured after the supernatant water was removed. The samples were dried at 120 °C until a constant weight was obtained. The water content was obtained using the following formula:

TE = ((M0 − M1)/M0) × 100

With

M0: Mass of wet sediment

M1: Mass of dry sediment

The total organic carbon (TOC) content of the sediments was determined according to the method described by Ref. [19]. Samples for chemical analyses were dried in an oven at a temperature of 60 °C for a period of 24 h. This was within 24 h of their collection. The 63 μm particle sizes were obtained by grinding the dried samples in an agate mortar. The XRD mineralogical analyses were performed at the Geosciences Laboratory at the Botswana International University of Science and Technology. The XRD diffractogram program was applied to ten representative samples. The XRD data were analyzed for phase identification and quantitative phase analysis using EVA and MATCH3. At the ALS Geochemistry Laboratory in Edenvale-Johanesburg, geochemical analyses were carried out. By using a Braun S4 Pioneer Rhodium (Rh) anode tube and an X-ray fluorescence spectrometer, the concentrations of the key elements SiO_2_, TiO_2_, MgO, CaO, MnO, Na_2_O, K_2_O, P2O5, and Fe_2_O_3_ were examined. When determining relative sensitivities, the detection thresholds are roughly 0.01%. Perkin Elmer Elan 9000 inductively coupled plasma-mass spectrometry carried out the analysis of the trace elements (Ba, Ni, Zn, Pb, Cd, Cu, V, and Co). The digestion was done with two diacid mixtures (HCl + HClO_4_) and (HNO_4_ + HCl). The validation of the results was checked by using the blank method with three certified reference materials (INTL 15-23810, DUP-17-41709, and BLANK-17-28360). This method is estimated to be accurate to within 5%. The results obtained from the geochemical analyses were used to calculate the coefficient of variation (Qi). This was obtained by comparing the concentrations of elements present in the EML with the average concentrations of the Mfoumou and Bengo'o rivers, taken as detrital references (rd).

Qi = Ci/rdi

With

rd = (Cmbe + CmNf)/2

CmBe: average concentration of element i in the Bengo'o river

CmNf: average concentration of element i in the Mfoumou River

We will have Qi < 1: depletion, Q = 1: no variation, and Qi > 1: enrichment.

Statistical analyzes were performed using Xlstat 2014 software. They were applied to data of the majors and traces elements, the values of coefficient of variation, and the physicochemical of the sediments. This allowed obtaining Pearson correlation matrices at p < 0.05. Also Hierarchical Cluster Analysis (HCA) was done. Prior to HCA, z scale transformation was used to normalize all data.

## 4 Results

### 4.1 Description of the samples

**Fig. 2:**
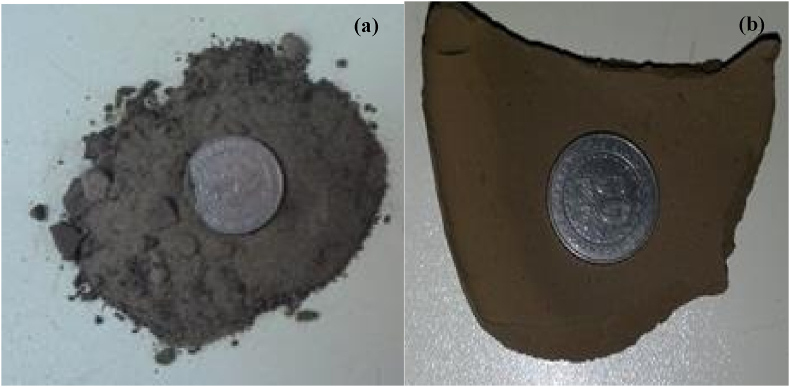
Sediment colors (a) grey colour characteristic of the samples from Mfoumou, Bengo'o, and the eastern and southern parts of the EML.

The colors, textures, and contents of the sediments studied are summarized in Table 1. These sediments are characterized by two main colors: grey (Fig. 2a) and brown (Fig. 2b). The former concerns all samples from the Mfoumou and Bengo'o rivers (NF1, NF2, NF3, NF4, NF5, NF6, NF7, Be1, Be2, Be3, and Be4) and those from the eastern (La1, La2, and La3) and southern (La9 and La10) parts of the EML. The second characterizes those from the western (La6, La7, and La8) and central (La4 and La4) parts of the EML and samples NF7 from the Mfoumou stream. The textures are coarse and fine. A coarse texture characterizes samples taken from Mfoumou and Bengo’o streams. As well as those collected in the eastern and southern parts of the lake. On the other hand, fine texture is observed in the sample from the central and western parts of the lake. All the collected samples are characterized by the presence of plant debris. However, the presence of laterite (NF5) and shell (La1, La2, La3, and La9) debris is also reported (Table 1).

**Table 1:** Description of the EML, Mfoumou and Bengo'o samples.

Sites	samples	Textures	Colors	Contains
Mfoumou	NF1	Coarse	2,5Y6/3 yellowish brown	Presence of plant material
	NF2	Coarse	2,5Y6/3 yellowish brown	Presence of plant material
	NF3	Coarse	2,5Y6/3 yellowish brown	Presence of plant material
	NF4	Coarse	10YR6/4 yellowish brown	Presence of plant material
	NF5	Coarse	10YR7/4 very light brown	Presence of plant and laterite debris
	NF6	Coarse	2,5Y6/3 yellowish brown	Presence of plant material
	NF7	Coarse	10YR7/4 yellowish brown	Presence of plant material
Bengo’o	Be1	Coarse	10YR4/1 dark grey	Presence of plant material
	Be2	Coarse	10YR7/3 very light brown	Presence of plant material
	Be3	Coarse	10YR7/3 very light brown	Presence of plant material
	Be4	Coarse	2,5Y6/3 yellowish brown	Presence of plant material
LME	La01	Coarse	10YR4/1 dark grey	Presence of plant and shell debris
	La02	Coarse	10YR4/1 dark grey	Presence of plant and shell debris
	La03	Coarse	10YR4/1 dark grey	Presence of plant and shell debris
	La04	fine	10YR7/3 very light brown	Presence of plant material
	La05	fine	10YR7/3 very light brown	Presence of plant material
	La06	fine	10YR7/3 very light brown	Presence of plant material
	La07	fine	10YR7/3 very light brown	Presence of plant material
	La08	fine	10YR7/3 very light brown	Presence of plant material
	La09	Coarse	2,5Y6/3 yellowish brown	Presence of plant and shell debris
	La10	Coarse	2,5Y6/3 yellowish brown	Presence of plant material

### 4.2 Physicochemical parameters

#### 4.2.1 Physicochemical parameters of the water

The values of the physicochemical parameters of the waters are presented in Table 2. In the EML, the pH values vary between 7.57 (La1) and 8.14 (La9), with an average value of 7.87. The average Eh values are −36.80 mV. The maximum value (−51 mV) is observed in sample La9, while the minimum value (−13.00 mV) is observed in sample La1. The temperature oscillates between 25.00 °C (La6) and 28.00 °C (La7). Its average value is 26.49 °C. The conductivity oscillates between 85.3 and 114.80 S/cm, with an average value of 102.29 S/cm. Turbidity values range from 20.73 to 62.0 NTU. Dissolved oxygen values range from 2.3 (La5) to 4.34 mg/L (La7). The salinity value for all samples at this site is equal to 0.01 mg/L. In the Mfoumou and Bengo'o streams, the pH values vary between 5.69 and 7.74, with an average value of 7.19. The maximum value of Eh is 91 mV, while the minimum value is −10.00 mV. Temperatures vary between 24.30 and 25.60 °C. There are presented in Table 2. In the EML, the pH values vary between 7.57 (La1) and 8.14 (La9), with an average value of 7.87. The average Eh values are −36.80 mV. The maximum value (−51 mV) is observed in sample La9, while the minimum value (−13.00 mV) is observed in sample La1. The temperature oscillates between 25.00 °C (La6) and 28.00 °C (La7). Its average value is 26.49 °C. The conductivity oscillates between 85.3 and 114.80 μS/cm, with an average value of 102.29 μS/cm. Turbidity values range from 20.73 to 62.0 NTU. Dissolved oxygen values range from 2.3 (La5) to 4.34 mg/L (La7). The salinity value for all samples at this site is equal to 0.01 mg/L. In the Mfoumou and Bengo'o streams, the pH values vary between 5.69 and 7.74, with an average value of 7.19. The maximum value of Eh is 91 mV, while the minimum value is −10.00 mV. Temperatures vary between 24.30 and 25.60 °C. Conductivity varies between 47.30 and 217.00 μS/cm. Turbidity values range from 6.75 to 28.78 NTU. Dissolved oxygen values range from 2.40 to 8.90 mg/L (La7). The salinity value for all samples at this site is equal to 0.01 mg/L.

**Table 2:** Physical parameters of the water.

Sites	Parameters	pH	Eh	T	Cs	Turb	OD	Sal
	Units		(mV)	°C	(μS/cm)	(NTU)	(mg/L)	(mg/L)
LME	La1	7.57	−13	26.2	85.3	20.73	2.6	0.01
	La2	7.74	−30	25	94.9	21.72	2.6	0.01
	La3	7.77	−31	26.7	96.7	20.64	2.6	0.01
	La4	7.86	−36	25.4	102.2	26.63	2.8	0.01
	La5	7.88	−38	26.7	102.9	36.08	2.3	0.01
	La6	7.95	−43	25	102.2	31.51	2.4	0.01
	La7	7.95	−42	28	114.8	26.25	4.34	0.01
	La8	7.89	−39	27.4	118.9	62	2.7	0.01
	La9	8.14	−51	27.3	103	40.38	2.7	0.01
	La10	7.9	−45	27.2	102	44.1	2.6	0.01
	Mean	7.87	−36.8	26.49	102.29	33	2.76	0.01
Mfoumou	NF2	7.77	36	24.6	118.9	15.47	2.4	0.01
	NF3	5.69	91	25.6	116.5	6.75	2.8	0.01
	NF4	7.45	86	25.4	115.2	14.25	2.6	0.01
	NF5	7.62	87	24.9	120.27	15.27	2.7	0.01
	NF6	7.74	54	25.2	198.7	17.8	2.8	0.01
	NF7	7.54	−10	25.3	217	28.78	3.4	0.01
	Mean	7.19	45.22	25.54	147.33	16.81	2.82	0.01
Bengo'o	Be2	7.1	15	25.2	84	13.2	8.9	0.01
	Be3	7.2	17	25.1	87	13.8	8.5	0.01
	Be4	7.42	−10	24.3	47.3	12.71	3	0.01
	Mean	7.18	8.75	24.78	74.93	13.53	7.78	0.01

#### 4.2.2 Physicochemical parameters of sediments

Table 3 summarizes the values of the physicochemical parameters of the sediments. In the EML, the water content varies between 12 and 75%. The sediments are relatively rich in organic carbon. Indeed, the proportions of TOC range from 2.08 to 12.65%. The pH varies between 5.14 and 6.95. The redox potential is between −48 and −5 mV. The conductivity varies from 85.30 to 318.9 μCs. Temperature values range from 25 to 27.4 °C. In the Mfoumou stream, the values of TE, TOC, pH, Eh, Cs, and T are respectively between 18 and 57%, 3.68 and 10.82%, 5.35 and 7.5, −25.53 and 98 mV, 63, 37, and 369.33 μCs and 26.96 and 28.7. In Bengo'o, the water content values range from 16 to 67%. TOC values range from 7.07 to 8.21%. The pH values of these sediments vary between 5.67 and 7.74. The redox potential varies between −39.66 and 86.27 mV. The conductivity values range from 47.20 to 84.60 μCs. Temperature values range from 27.46 to 28.20 °C.

**Table 3:** Physical parameters of sediments.

Sites	Samples	TE (%)	pH	Eh (mV)	Cs (μS/cm)	T (°C)	TOC (%)
LME	La01	0.34	5.57	−18	85.3	26.2	9.25
	La02	0.43	6.74	−35	91.9	25	8.32
	La03	0.42	5.77	−30	86.7	26.7	10.46
	La04	0.75	6.86	−29	202.2	25.4	12.65
	La05	0.65	6.88	−43	202.9	26.7	11.04
	La06	0.6	6.95	−48	202.2	25	8.35
	La07	0.67	6.95	−42	314.8	28	8.1
	La08	0.75	6.89	−39	318.9	27.4	10.8
	La09	0.14	5.14	−10	303	27.3	2.63
	La10	0.12	5.9	−5	347	27.2	2.08
	Mean	0.49	6.37	−29.9	215.49	26.49	8.37
Mfoumou	NF1	0.5	7.5	−25.53	369.33	27.53	8.18
	NF2	0.43	5.43	94.23	83.16	28.4	8.98
	NF3	0.36	6.98	3.6	152.56	26.9	5.69
	NF4	0.21	5.44	93.43	37	27.46	7.06
	NF5	0.19	6.83	13.33	55.8	26.96	4.31
	NF6	0.18	6.2	49.77	76.36	28.7	3.68
	NF7	0.57	5.35	98.63	181.5	27.46	10.42
	Mean	0.35	6.25	46.78	136.53	27.63	6.9
Bengo’o	Be1	0.29	5.67	86.27	84.6	28.2	8.21
	Be2	0.16	7.22	7.1	73.56	27.73	8.21
	Be3	0.67	5.77	74.6	47.2	28.36	7.07
	Be4	0.17	7.74	−39.66	75.3	27.63	7.9
	Mean	0.32	6.6	32.08	70.17	27.98	7.85

### 4.3 Mineralogy

**Fig. 3:**
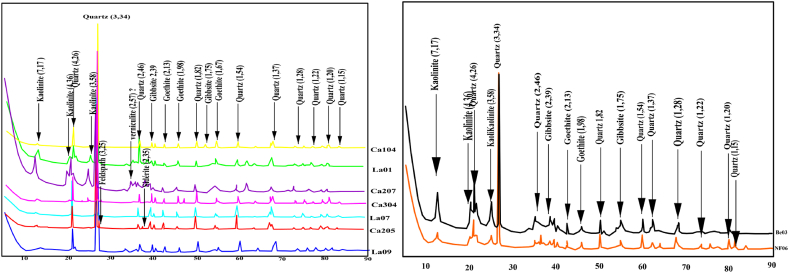
Diffractograms of analyzed samples (a) EML, (b) Mfoumou and Bengo'o.

The mineralogical composition of the sediments from the three sites is characterized by the presence of quartz, kaolinite, gibbsite, and goethite (Table 4). Quartz is the most represented mineral. It is highly abundant in all samples. Kaolinite is the second most abundant mineral present. It is high in samples La3 and La4; well represented in samples La1, La4, and NF7; and weakly represented in the other samples. The remaining minerals (gibbsite and goethite) occur only in trace amounts. Siderite was only observed in the lake (Fig. 3).

**Table 4:** Mineralogical composition of the overall sediment fractions.

Sites	LME								Bengo'o	Mfoumou
Samples	La01	La04	La07	La09	Ca104	Ca205	Ca207	Ca304	Be 4	NF7
Quartz	++++	++++	++++	++++	++++	++++	++++	++++	++++	++++
Kaolinite	+++	+++	+++	–	–	+++	+++	+++	+	+++
Feldspaths	+	+	+	–	–	+	–	–	–	+
Goethite	+	–	+	+	+	+	+	+	+	+
Siderite	–	–	–	–	–	+	+	+	–	–
Gibbsite	++	++	++	–	++	++	++	++	++	++

++++ very abundant. +++ abundant. ++ represented. +poorly represented. – no identified.

### 4.4 Geochemistry

#### 4.4.1 Major elements

The major element contents of the sediments from the three sites are presented in Table 5. This table shows that in the EML, the SiO_2_ content varies between 60.44 and 89.57%. Al_2_O_3_ contents range from 6.55 to 18.17%. Fe_2_O_3_ varies from 6.21 to 1.15%. K_2_O values are in the 218 range 0.99–2.34. TiO_2_, CaO, Na_2_O, MnO, MgO, and P_2_O_5_ values are below unity. The loss on ignition (LOI) varies between 2.08 and 11.04%. In the Mfoumou River, SiO_2_ varies from 69 to 82.32%. Al_2_O_3_ concentrations range from 7.51 to 11.58%. Fe_2_O_3_ and K_2_O values range from 2.38% to 5.30% and 1.22 to 2.12%, respectively. The contents of TiO_2_, CaO, Na_2_O, MnO, MgO, and P_2_O_5_ do not exceed 0.5%. The loss on ignition varies from 3.68 to 10.42%. In the Bengo'o stream, SiO2 values range from 74.11 to 75.94%. Al_2_O_3_ values range from 10.00 to 10.85%. Fe2O3 and K2O contents are between 3.70 and 4.40 and 1.09 and 2.27%, respectively. The contents of TiO2, CaO, Na_2_O, MnO, MgO, and P_2_O_5_ are less than 0.5%. The LOI values range from 7.07 to 8.21%. The values of the Mn/Fe ratios are lower than 0.60 in all the samples.

**Table 5:** Distribution of major elements in sediments.

Sites	LME											Mfoumou								Bengo’o				
Samples	La01	La02	La03	La04	La05	La06	La07	La08	La09	La10	Mean	NF1	NF2	NF3	NF4	NF5	NF6	NF7	Mean	Be1	Be2	Be3	Be4	Mean
SiO_2_	70.21	71.44	70.54	66,00	60.44	65.17	64,00	64.25	81.07	83.57	70.61	75.58	75.21	77.06	76.69	80.5	82.3	68,00	76.6	75.24	74.4	74,00	74.1	75.16
TiO_2_	0.34	0.32	0.31	0.38	0.54	0.28	0.29	0.31	0.21	0.19	0.32	0.11	0.2	0.18	0.19	0.21	0.22	0.4	0.22	0.26	0.24	0.27	0.28	0.26
Al_2_O_3_	12.27	12.24	12.11	14.23	18.17	16.43	15.84	15.91	9.21	6.55	13.3	10.11	9.78	9.21	8.83	9.06	7.51	11.58	9.53	10.16	10.7	10,00	10.9	10.42
Fe_2_O_3_	4.26	4.73	3.02	5.23	5.83	5.91	6.21	5.21	2.37	1.15	4.5	2.38	3.31	4.61	4.61	3.83	4,00	5.3	4.01	4.03	3.7	4.4	4.05	4.05
MnO	0.1	0.11	0.1	0.17	0.19	0.21	0.24	0.24	0.05	0.04	0.15	0.08	0.12	0.1	0.07	0.09	0.1	0.24	0.11	0.05	0.06	0.08	0.09	0.07
MgO	0.18	0.16	0.17	0.15	0.2	0.21	0.17	0.19	0.09	0.1	0.16	0.11	0.14	0.15	0.1	0.21	0.1	0.18	0.14	0.09	0.11	0.1	0.14	0.11
CaO	0.09	0.09	0.1	0.12	0.13	0.13	0.16	0.19	0.05	0.04	0.11	0.17	0.1	0.1	0.12	0.07	0.15	0.11	0.12	0.11	0.09	0.06	0.09	0.09
K_2_O	2.25	1.62	1.25	1.14	2.27	2.22	1.97	2.34	1.14	0.99	1.72	1.49	1.22	2.12	1.54	1.92	1.19	1.99	1.59	1.23	1.09	1.71	2.27	1.58
Na_2_O	0.27	0.35	0.17	0.16	0.27	0.28	0.29	0.29	0.16	0.18	0.24	0.18	0.14	0.41	0.34	0.24	0.19	0.34	0.27	0.28	0.19	0.24	0.35	0.27
P_2_O_5_	0.18	0.19	0.15	0.22	0.31	0.25	0.2	0.25	0.08	0.05	0.19	0.08	0.18	0.12	0.12	0.09	0.13	0.16	0.12	0.09	0.09	0.11	0.12	0.1
LOI	9.5	7.3	10.46	12.65	10.01	8.45	8.1	9.71	2.63	2.08	8.37	7.08	8.98	4.69	6.06	4.01	3.6	10.42	7.07	7.01	8.21	7.07	7.9	7.85
Total	100.3	98.54	98.88	101,00	99.03	100.1	98.02	99.14	98.8	97.23	99.1	98.08	100,00	98.5	99,00	98.7	100,00	98.99	99.1	98.8	99,00	98.1	100,00	99.03
Mn/Fe	0.02	0.02	0.03	0.03	0.03	0.04	0.04	0.05	0.02	0.03	0.03													

#### 4.4.2 Trace elements

The trace element contents in ppm of the sediments studied are presented in Table 6. These results show that in the EML, Ba values range from 745 to 978.22 ppm. Pb values range from 3.92 to 19.5 ppm. Zn ranges from 31.67 to 64.68 ppm. Co and Ni belong to the ranges [12.98–18.44] and [47.27–65.66], respectively. V contents range from 78.92 to 135.91 ppm. Cd contents range from 0.07 to 0.19 ppm. Cu ranges from 13.12 to 51.22 ppm.

**Table 6:** Distribution of trace elements in sediments.

Sites	LME											Mfoumou								Bengo'o				
Samples	La01	La02	La03	La04	La05	La06	La07	La08	La09	La10	Mean	NF1	NF2	NF3	NF4	NF5	NF6	NF7	Mean	Be1	Be2	Be3	Be4	Mean
Ni	29.87	30.21	30.28	35.25	36.24	35.21	34.25	36.24	21.02	19.08	30.77	31.25	30.21	32.24	36.54	31.02	21.05	36.47	31.25	18.24	19.72	17.89	22.23	19.52
Co	16.2	15.75	16.51	16.76	18.44	17.6	16.42	16.31	14.43	12.98	16.14	12.19	16.5	17.26	18.21	15.98	12.16	18.74	15.86	11.44	12.59	14.18	13.08	12.82
Zn	53.42	53.24	53.27	60.92	63.18	63.91	64.68	63.95	51.19	31.67	55.94	53.44	56.52	61.92	63.84	61.66	51.05	64.58	59	52.69	54.44	61.76	59.46	57.09
Pb	9	6.75	6	11.25	11.25	15.75	16.5	19.5	5.9	3.92	10.58	6.53	4.88	5.85	6.75	7.5	9	4.65	6.45	4.05	5.85	6.68	6.75	5.83
Cd	0.1	0.11	0.08	0.18	0.19	0.18	0.19	0.18	0.08	0.07	0.14	0.14	0.11	0.16	0.11	0.16	0.17	0.23	0.15	0.06	0.08	0.07	0.06	0.07
Cu	31.11	28.35	28.98	51.22	42.24	42.36	44.25	43.21	15.25	13.12	34.01	26.22	27.94	34.25	32.21	31.02	21.05	28.25	28.71	13.24	15.24	14.45	19.87	15.7
Ba	851.58	849.33	856.24	953.24	978.22	968.54	954.24	945.22	778.54	745.21	888.04	789.65	987.22	874.21	789.58	874.68	785.54	874.69	853.7	785.33	785.2	698.3	854.3	780.78
V	118.68	120.94	117.18	133.68	135.91	133.69	131.92	125.9	78.92	93	118.98	108.75	118.5	94.52	86.27	93.16	99.04	92.66	98.98	99.67	86.15	79.43	78.18	85.86

In the Mfoumou River, Ba values range from 874.68 to 987.22 ppm. Pb values range from 4.88 to 9 ppm. Zn ranges from 51.05 to 64.68 ppm. Co and Ni belong to the ranges [12.16–18.74] and [21.05–36.47], respectively. V contents range from 92.99 to 118.50 ppm. Cd contents range from 0.11 to 0.23 ppm. Cu ranges from 21.05 to 34.25 ppm. 237.

In the Bengo'o River, Ba values range from 698.3 to 854.3 ppm. Pb values range from 4.05 to 6.75 ppm. Zn ranges from 54.44 to 61.76 ppm. Co and Ni belong to the ranges [11.44–14.18] and [17.89–22.23], respectively. V contents range from 78.18 to 99.67 ppm. Cd contents range from 0.06 to 0.08 ppm. Cu ranges from 14.45 to 19.87 ppm. 4.5.

The values of the coefficient of variation (Qi) of the different elements are presented in Table 7. These results show that the lowest values of this parameter are observed in the samples taken toward the outlet. The highest values are observed in the central and western parts of the lake. The Si values are lower than 1 in all samples except samples La09 and La10. Ca values are, on average, equal to 1. However, the highest values were obtained in the central and western parts of the lake. The average value of Ti is equal to 1. The other elements (Al, Fe, Mn, Mg, K, Na, P, Ni, Co, Zn, Pb, Cd, Cu, Ba, and V) have average Qi values greater than 1.

**Table 7:** Distribution of coefficient of variation values in the EML.

Elements	La01	La02	La03	La04	La05	La06	La07	La08	La09	La10	Mean
SiO_2_	0.93	0.94	0.93	0.87	0.8	0.86	0.84	0.85	1.07	1.1	0.9
TiO_2_	0.89	0.79	1.29	0.87	0.84	1.17	1.1	1.29	0.88	0.79	1
Al_2_O_3_	1.23	1.23	1.21	1.43	1.82	1.65	1.59	1.59	0.92	0.66	1.3
Fe_2_O_3_	1.06	1.17	0.75	1.3	1.45	1.47	1.54	1.29	0.59	0.29	1.7
MnO	1.11	1.22	1.11	1.89	2.11	2.33	2.67	2.67	0.56	0.44	1.5
MgO	1.44	1.28	1.36	1.2	1.6	1.68	1.36	1.52	0.72	0.8	1.3
CaO	0.86	0.86	0.95	1.14	1.24	1.24	1.52	1.81	0.48	0.38	1
K_2_O	1.42	1.02	0.79	0.72	1.43	1.4	1.24	1.48	0.72	0.62	1.1
Na_2_O	1	1.3	0.63	0.59	1	1.04	1.07	1.07	0.59	0.67	1.1
P_2_O_5_	1.64	1.73	1.36	2	2.82	2.27	1.82	2.27	0.73	0.45	1.7
Ni	1.18	1.19	1.19	1.39	1.43	1.39	1.35	1.43	0.83	0.75	1.2
Co	1.13	1.1	1.15	1.17	1.29	1.23	1.15	1.14	1.01	0.91	1.1
Zn	0.92	0.92	0.92	1.05	1.09	1.1	1.11	1.1	0.88	0.55	1
Pb	1.47	1.1	0.98	1.83	1.83	2.57	2.69	3.18	0.96	0.64	1.7
Cd	0.91	1	0.73	1.64	1.73	1.64	1.73	1.64	0.73	0.64	1.3
Cu	1.4	1.28	1.31	2.31	1.9	1.91	1.99	1.95	0.69	0.59	1.5
Ba	1.04	1.04	1.05	1.17	1.2	1.19	1.17	1.16	0.95	0.91	1.1
V	1.28	1.31	1.27	1.45	1.47	1.45	1.43	1.36	0.85	1.01	1.3

### 4.5 Statistical analyses

#### 4.5.1 Pearson correlation matrix

The correlation matrix highlighting the relationship between the chemical elements and the physicochemical parameters is presented in Table 8. This one presents significant correlations between the different parameters (p < 0.05). There are significant negative correlations between Si and other major elements (r > −0.70), except with Ti (r = −0.30). Significant correlations at p < 0.05 are observed between Al oxide and the entire studied element (r > 0.70), except with Na oxide (r = 0.43). Nutrients (P, Ba, Zn, Cu, and Co) correlate significantly (r > 0.75). The elements associated with redox conditions (Fe and Mn) show high correlation (r = 0.90). Similarly, some alkalines (Mg and K) have a strong correlation (r = 0.82). The Eh correlates significantly with all elements analyzed. Similarly, a significant correlation between pH and chemical elements is observed, except with Ti, K, Na, and Co. TOC correlate significantly with Mg (r = 0.72), P (r = 0.68), Ni (r = 0.76), Co (r = −0.80), V (r = 0.69) and Ba (r = 0.60).

**Table 8:** Correlation matrix illustrating relationships between elements and physical parameters of sediments.

																						
Variables	SiO_2_	TiO_2_	Al_2_O_3_	Fe_2_O_3_	MnO	MgO	CaO	K_2_O	Na_2_O	P_2_O_5_	Ni	Co	Zn	Pb	Cd	Cu	Ba	V	pH	Eh	T	TOC
SiO_2_	**1.00**																					
TiO_2_	−0.30	**1.00**																				
Al_2_O_3_	**−0.89**	0.27	**1.00**																			
Fe_2_O_3_	**−0.77**	0.26	**0.92**	**1.00**																		
MnO	**−0.87**	0.44	**0.91**	**0.92**	**1.00**																	
MgO	**−0.72**	0.41	**0.82**	**0.73**	**0.71**	**1.00**																
CaO	**−0.90**	0.56	**0.92**	**0.86**	**0.97**	**0.75**	**1.00**															
K_2_O	**−0.75**	0.37	**0.74**	0.63	**0.71**	**0.87**	**0.72**	**1.00**														
Na_2_O	−0.59	−0.07	0.43	0.50	0.59	0.44	0.44	0.63	**1.00**													
P_2_O_5_	**−0.79**	0.17	**0.95**	**0.87**	**0.85**	**0.78**	**0.84**	**0.73**	0.42	**1.00**												
Ni	**−0.75**	0.32	**0.91**	**0.85**	**0.85**	**0.73**	**0.86**	0.63	0.27	**0.96**	**1.00**											
Co	−0.52	0.20	**0.71**	0.60	0.46	**0.66**	0.54	0.39	−0.08	**0.71**	**0.75**	**1.00**										
Zn	**−0.82**	0.40	**0.96**	**0.93**	**0.94**	**0.75**	**0.94**	**0.71**	0.40	**0.91**	**0.88**	0.57	**1.00**									
Pb	**−0.82**	0.45	**0.87**	**0.88**	**0.97**	**0.68**	**0.94**	**0.76**	0.54	**0.84**	**0.85**	0.39	**0.93**	**1.00**								
Cd	**−0.86**	0.11	**0.94**	**0.94**	**0.90**	0.63	**0.85**	0.61	0.49	**0.89**	**0.84**	0.58	**0.92**	**0.87**	**1.00**							
Cu	**−0.72**	0.31	**0.81**	**0.85**	**0.83**	0.49	**0.81**	0.45	0.22	**0.78**	**0.87**	0.60	**0.82**	**0.85**	**0.87**	**1.00**						
Ba	**−0.77**	0.37	**0.93**	**0.91**	**0.91**	0.64	**0.91**	0.58	0.27	**0.91**	**0.93**	0.63	**0.96**	**0.91**	**0.92**	**0.92**	**1.00**					
V	**−0.85**	0.18	**0.97**	**0.89**	**0.85**	**0.76**	**0.87**	0.63	0.35	**0.95**	**0.95**	**0.75**	**0.90**	**0.82**	**0.93**	**0.86**	**0.91**	**1.00**				
pH	**−0.65**	0.22	**0.80**	**0.90**	**0.86**	0.60	**0.80**	0.42	0.47	**0.76**	**0.79**	0.45	**0.82**	**0.79**	**0.82**	**0.77**	**0.82**	**0.82**	**1.00**			
Eh	**0.76**	−0.31	**−0.90**	**−0.90**	**−0.85**	**−0.87**	**−0.82**	**−0.71**	−0.54	**−0.85**	**−0.78**	**−0.67**	**−0.87**	**−0.75**	**−0.82**	−0.62	**−0.78**	**−0.82**	**−0.81**	**1.00**		
T	−0.24	0.25	0.02	−0.10	0.13	−0.15	0.22	−0.01	0.01	−0.17	−0.19	−0.44	0.13	0.14	0.04	−0.04	0.04	−0.07	−0.03	0.14	**1.00**	
TOC	−0.55	0.24	0.60	0.79	0.64	0.72	0.73	0.42	−0.09	**0.68**	**0.76**	**0.80**	0.48	0.46	0.49	0.64	0.60	**0.69**	0.26	−0.37	−0.27	**1.00**

#### 4.5.2 Hierarchical cluster analysis (HCA)

**Fig. 4:**
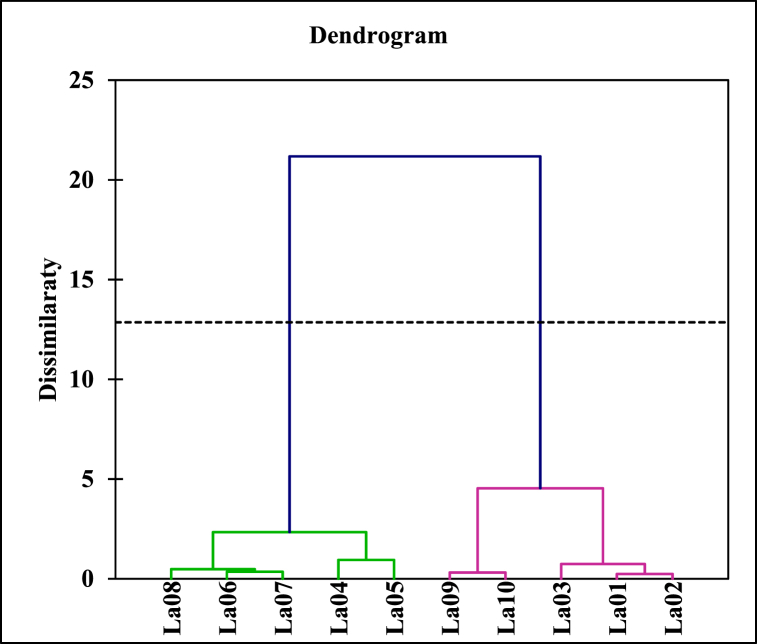
Dendrogram illustrating the relationships between the different EML samples.

HCA highlighting the variation of Qi is shown in Fig. 4. This figure includes two clusters (cluster 1 and cluster 2). Cluster 1 includes samples from the central and western parts of the lake (La04, La05, La06, La07, and La10). They characterize the sample with the highest Qi values. Cluster 2 includes samples from the eastern and southern parts of the lake. They have the lowest Qi values.

## 5 Discussion

### 5.1 Redox and paleoredox conditions

Studies on redox conditions in the aquatic environment aim to determine the distribution of oxidizing agents along depositional and diagenesis gradients, on the one hand, and the biogeochemical processes that control this distribution, on the other [27]. There are several markers of redox conditions, including dissolved oxygen content, redox potential, organic matter content, Fe and Mn concentrations, and Mn/Fe ratios, etc. DO levels, Eh, and pH are the markers used to assess the redox conditions of the water column. According to the work of [28], values of OD > 2 mg/L reflect an oxic environment; 2 > OD > 0.2 mg/L, a suboxic environment; OD < 2 mg/L, an anoxic environment; and OD = 0, an euxinic environment. In the present study, all the samples analyzed had OD values above 2 mg/L, reflecting an oxic environment. The pH values illustrate that these waters are neutral to basic (7 < pH < 8). The high values of this parameter, despite the acidic nature of the bedrock, would be linked to a high concentration of organic matter in them, as illustrated by the high turbidity levels (Turb > 1 NTU) in them. Indeed, the work of [18] has shown that organic matter is one of the factors that lead to an increase in pH in an environment. The Eh values illustrate that these waters are decreasing. According to Ref. [29], the reducing character of these waters is linked to the degradation of organic matter. In sediments, redox conditions have been assessed not only through Fe and Mn concentrations but also through pH and Eh values. For several authors [[29], [30], [31]] Fe and Mn are very sensitive to the redox conditions of the environment. However, the oxidation of Fe is faster than that of Mn [32]. Thus, the Mn/Fe ratio is higher in an oxic environment than in anoxic environment. In the present study, the Mn/Fe ratio values are below 0.4. This illustrates that the sediments studied are anoxic [12]. Similarly, the pH values are below 7 in all samples (Table 3). This result illustrates that they are acidic, reflecting the nature of the basement (granitic and gneissic). Similarly, Eh values are much lower than in the water column, illustrating their more reductive character than surface waters. The rapid transition from oxic conditions (surface water) to anoxic conditions (bottom sediments) would be linked to high temperatures which activate the action of microorganisms responsible for the mineralization of organic matter [12,27,33]. This phenomenon would lead to the precipitation of any condition-sensitive elements in the bottom sediments. This explains the enrichment of Fe and Mn in the EML (Qi > 1). This rapid consumption of oxygen would also explain the accelerated eutrophication observed in this lake, as reported by Refs. [26,34]. Also, the iron enrichment in the lake and the reductive conditions prevailing there would be at the origin of the precipitation of siderite, which is present only in the mineralogical procession of the lake.

### 5.2 Productivity and paleoproductivity

Several elements are associated with lake productivity, notably phosphorus, barium, nickel, copper, and cadmium [35,36]. Phosphorus is enriched in the EML (Qi > 1). The significant correlation between phosphorus and TOC (r = 0.68) illustrates that phosphorus is associated with organic matter. Several authors [12,27,33,35] have shown that the authigenic phosphorus present in aquatic environments comes mainly from the mineralization of organic matter. Indeed, the work of [27] has shown that phosphorus comes from the degradation of organic matter by bacteria at the water-sediment interface, especially under anoxic conditions. According to Ref. [37], phosphorus is released in the form of phosphate ions. These ions return to the water column and then precipitate. This constitutes the productivity of the phytoplankton. The significant correlation between P and Fe (r = 0.85) illustrates that the enrichment of phosphorus is also linked to that of iron. Indeed, several studies [38,39] have shown that reducing conditions favor the absorption of P on Fe and then their deposition in the bottom sediments.

Barium, nickel, copper, zinc, nickel, cadmium, and vanadium are enriched in the EML (Qi > 1). Also, these elements correlate significantly with each other, especially with phosphorus and TOC (Table 3). This result illustrates that these elements are also associated with organic matter content. According to several authors [40,41], metallic elements associate with organic matter to form organometallic complexes. Thus, the degradation of organic matter by aquatic microorganisms under reducing conditions increases their concentration in sediments. Silica and calcium are generally cosmic elements. These elements enter into the constitution of the skeleton of aquatic organisms. However, silica has a Qi value of 0.91, which proves that, despite the presence of biogenic silica associated with the presence of cosmic elements (snail shell), it is depleted in this environment. This depletion would be linked to the predominance of the fine fraction in this environment. It is mainly associated with the process of replacement (desilicification) of silica by other elements such as Fe_2_O_3_ and Al_2_O_3_, as shown by the strong negative correlations between silica and the rest of the elements (Table 8). Also, the presence of important proportions of kaolinite and iron carbonates, notably siderite, which is only present in the lake, seems to support this hypothesis. Calcium is characterized by very low concentrations (Table 5). These low concentrations would be linked to the intense alteration that the source rocks of these sediments undergo [21]. Indeed, the work [42] showed that chemical alteration is accompanied by the departure of alkalis such as K, Na, Mg, and Ca. Also, the Qi value of Ca is approximately equal to 1. This illustrates that there is neither depletion nor enrichment in the EML. Thus, the calcium in the lake would have a terrigenous origin. The absence of the formation of authigenic Ca (calcite) would be linked to the low pH values in the water and sediments. Indeed, according to Ref. [38], the precipitation of calcium is observed when the pH value of the water is below 8.2. In the present study, the pH values are lower than this value (Table 2, Table 3).

### 5.3 Spatial distribution of diagnostic processes

The diagenesis processes that take place in aquatic environments are influenced by the physicochemical parameters of the environment. Thus, within the same watercourse, different processes could take place. The bottom-up hierarchical classification (Fig. 4) shows two groups of samples, the first group consisting of samples from the central and western parts of the lake, and the second group consisting of samples from the eastern and southern parts of the lake. The samples from the first group have the highest Qi values. This illustrates that elemental enrichments would be higher in these parts (especially in the western part). Also, the sediments in these parts are characterized by a darker color (Fig. 2). According to the work of [18], this characteristic illustrates the degradation of organic matter. The work of [43] showed that the activity of microorganisms responsible for the decomposition of organic matter is accelerated under anoxic conditions. This suggests that the redox conditions are more reducing in this part of the lake. Thus, the important enrichment observed in these parts would be linked to these conditions, which would favor a more important biological activity and consequently an acceleration of the mineralization process of the matter in this part of the lake. The work [22] has indeed shown that the western part of the lake is the one most subjected to anthropic activity, which makes it the most affected by the accelerated eutrophication process observed in this lake in recent years.

## 6 Conclusion

The objective of the present work was to perform a geochemical assessment of the inorganic fraction of the EML sediments, determine the redox conditions in the water column and bottom sediments, identify the diagenetic processes taking place in this lake, and quantify the influence of these processes on the composition of the EML sediments. Sediments were collected from the EML and its two tributaries. Sediments collected from the tributaries were used as a detrital reference. There is a rapid consumption of oxygen, which is characterized by the transition from oxic conditions in the water column to anoxic conditions at the sediment surface. These conditions favor the precipitation of elements sensitive to redox conditions (Fe and Mn). The enrichment of iron leads to the formation of siderite. The mineralization of organic matter is the dominant early diagenesis process in this lake. It leads to the enrichment of phosphorus and trace metals (Ni, Co, Zn, Pb, Cd, Cu, Ba, and V) in the lake. Early diagenesis processes are more pronounced in the western part of the lake. The results found provide important information for the understanding of diagenesis phenomena that take place in the EML. However, the analysis of major anions and cations would provide additional information for a better understanding of this phenomenon. This study showed that early diagenesis plays an important role in the composition of aquatic sediments. Also, the processes associated with it are not the same over the entire extent of a watercourse.

## Author contribution statement

Daniel Florent Akono, Eric Bokanda Ekoko, Bertrant Bisse Salomon and Ekomane Emile: Conceived and designed the experiments; Performed the experiments; Analyzed and interpreted the data; Contributed reagents, materials, analysis tools or data; Wrote the paper.

Cedric Belinga Belinga, Renaud Menanga Tokouet, Marie-Diane Tonye, Estelle Diane Biani Nya, Leon Vital Ngono Anaba and Mouhamed Amin Nsangou: Contributed reagents, materials, Analyzed and interpreted the data; Wrote the paper.

Valentino Nzesseu Nandjou and Cedric Kouakam: Analyzed and interpreted the data; Wrote the paper.

## Data availability statement

Data included in article/sup. Material/referenced in article.

## Declaration of competing interest

The authors declare that they have no known competing financial interests or personal relationships that could have appeared to influence the work reported in this paper.
